# Comparative Analysis of ChatGPT and Google Gemini in Generating Patient Educational Resources on Cardiac Health: A Focus on Exercise-Induced Arrhythmia, Sleep Habits, and Dietary Habits

**DOI:** 10.7759/cureus.80771

**Published:** 2025-03-18

**Authors:** Nithin Karnan, Sumaiya Fatima, Palwasha Nasir, Lovekumar Vala, Rutva Jani, Nahir Montserrat Moyano

**Affiliations:** 1 Internal Medicine, K.A.P. Viswanathan Government Medical College, Tiruchirappalli, IND; 2 Internal Medicine, Jinnah Sindh Medical University, Karachi, PAK; 3 Internal Medicine, Emilio Aguinaldo College, Manila, PHL; 4 Internal Medicine, Shantabaa Medical College, Amreli, IND; 5 Internal Medicine, Chimanlal Ujamshibhai (CU) Shah Medical College and Hospital, Surendranagar, IND; 6 Internal Medicine, Universidad Nacional de Asuncion-Facultad de Ciencias Medicas, Asunción, PRY

**Keywords:** artificial intelligence, cardiovascular health, chatgpt, google gemini, patient education guide

## Abstract

Introduction: Patient education is crucial in cardiovascular health, aiding in shared decision-making and improving adherence to treatments. Artificial intelligence (AI) tools, including ChatGPT (OpenAI, San Francisco, CA) and Google Gemini (Google LLC, Mountain View, CA), are revolutionizing patient education by providing personalized, round-the-clock access to information, enhancing engagement, and improving health literacy. The paper aimed to compare the responses generated by ChatGPT and Google Gemini for creating patient education guides on exercise-induced arrhythmia, sleep habits and cardiac health, and “dietary habits and cardiac health.

Methodology: A comparative observational study was conducted evaluating three AI-generated guides: "exercise-induced arrhythmia," "sleep habits and cardiac health," and "dietary habits and cardiac health," using ChatGPT and Google Gemini. Responses were evaluated for word count, sentence count, grade level, ease score, and readability using the Flesch-Kincaid calculator and QuillBot (QuillBot, Chicago, IL) plagiarism tool for similarity score. Reliability was assessed with the modified DISCERN score. Statistical analysis was conducted using R version 4.3.2 (The R Core Team, R Foundation for Statistical Computing, Vienna, Austria).

Results: ChatGPT-generated responses had an overall higher average word count when compared to Google Gemini; however, the difference was not statistically significant (p = 0.2817). Google Gemini scored higher on ease of understanding, though this difference was also not significant (p = 0.7244). There were no significant differences in sentence count or average words per sentence. ChatGPT tended to produce more complex content for certain topics, whereas Google Gemini's responses were generally easier to read. Similarity scores were higher for ChatGPT across all topics, while reliability scores varied by topic, with Google Gemini performing better for exercise-induced arrhythmia and ChatGPT for sleep habits and cardiac health.

Conclusions: The study found no significant difference in ease score, grade score, and reliability between AI-generated responses for a cardiology disorders brochure. Future research should explore AI techniques across various disorders, ensuring up-to-date and reliable public information.

## Introduction

In the digital era, chatbots and artificial intelligence (AI) tools are significantly transforming various professional fields, including healthcare. These advanced technologies are reshaping how information is delivered and managed and are increasingly being utilized to enhance patient education [1]. Chatbots, in particular, are emerging as critical tools in providing medical information efficiently and effectively, offering personalized support at any time of day. This innovation is particularly relevant in the context of cardiovascular health, where patient education is essential for managing chronic conditions and promoting overall well-being.

Cardiovascular diseases are a leading cause of morbidity and mortality worldwide, underscoring the need for effective patient education to manage risk factors and improve health outcomes [2]. The connection between heart health and dietary habits is developing, with lifestyle adjustments, particularly in dietary choices, being the main strategy for handling risk factors related to heart and metabolic health. Therefore, it is crucial for patients to understand the effects of various diets on cardiovascular well-being to effectively prevent both initial and recurring cardiovascular diseases [3]. Thus, educating patients about these lifestyle factors is crucial, as these elements can significantly impact the progression and management of heart conditions [4].

Patient education is a vital component of healthcare and is a well-established factor that can lead to increased patient involvement in shared decision-making, better adherence to medications and treatments, higher levels of patient satisfaction, and ultimately, improved treatment results [5]. However, traditional methods of patient education, such as in-person consultations and printed materials, can be limited by accessibility issues and variability in information delivery.

The introduction of AI-driven chatbots such as ChatGPT (OpenAI, San Francisco, CA) and Google Gemini (Google LLC, Mountain View, CA) offers a novel approach to overcoming these limitations. These tools utilize natural language processing (NLP) to generate personalized educational content tailored to individual patient needs and preferences [6]. By providing on-demand, accessible information, these chatbots have shown effectiveness in influencing lifestyle behaviors. This personalized interaction can improve patient engagement and encourage healthier behaviors, potentially leading to better management of chronic conditions and enhanced adherence to treatment plans, thus not only saving healthcare professionals' time but also enhancing overall health literacy [7].

ChatGPT, an advanced language model created by OpenAI, has promising applications in public health. Leveraging its capacity to produce human-like text from extensive data, ChatGPT can assist individuals and communities in making well-informed decisions about their healthcare [8]. Gemini is a powerful AI model developed by Google's AI research division and has access to real-time data along with training on a vast amount of text and code data, equipping it with extensive knowledge [9].

Despite their potential, AI chatbots pose several challenges, including misinformation risks, biased training data, and plagiarism concerns. Furthermore, very few studies have directly compared AI models in generating patient education materials. 

Investigating the effectiveness of AI-generated educational materials is vital for understanding their impact. Of the AI-driven chatbots currently available, ChatGPT and Google Gemini are two of the most common, freely available, and widely used chatbots. Thus, this study aims to compare the efficacy of ChatGPT and Google Gemini in generating patient education guides on specific cardiovascular topics: exercise-induced arrhythmia, sleep habits, and dietary habits. By analyzing the content produced by these AI tools, we seek to evaluate their quality in terms of readability, comprehensiveness, and practical utility for patients. This comparative analysis will offer insights into how different AI systems contribute to patient education and highlight areas for enhancement in cardiovascular health information delivery.

Aims and objectives

Our study aims to compare ChatGPT and Google Gemini for creating patient education guides on exercise-induced arrhythmias, sleep habits and cardiac health, and dietary habits and cardiac health, focusing on readability, comprehension, and usability, to enhance the accessibility of crucial information for all readers.

## Materials and methods

This was a comparative observational original research study conducted in April 2024. As no human participants were involved, the study was deemed exempt from ethical approval.

Study tools

Three common diseases in cardiology, namely hypertension, arrhythmias, and ischemic heart diseases, were selected for analysis. Two AI tools, namely ChatGPT 3.5 (on 7^th^ April) and Google Gemini 1.0 Pro (on 7^th^ April), were chosen for generating patient education brochures. Each AI tool received three prompts: Question 1 (Q1): Make a patient education guide for exercise-induced arrhythmias; Q2: Make a patient education guide for sleep habits and cardiac health; and Q3: Make a patient education guide for dietary habits and cardiac health. The questions used were framed in a neutral way in order to avoid bias.

Each author generated one response in one chatbot to minimize the potential for any inadvertent influence on the responses. This prevented the models from being "fine-tuned" or "trained" to previous queries that would have been asked, as these chatbots continuously train, and data from each interaction are ingested to improve the machine learning model. The responses were collected in a Microsoft Word document (Microsoft Corp., Redmond, WA).

The generated responses underwent grading using three tools.

Firstly, the Flesch-Kincaid calculator was employed to assess word count and sentence count. The Flesch-Kincaid grade level is a widely used readability formula that assesses the approximate reading grade level of a text based on the average sentence length and word complexity. We chose this metric to assess the range of patient demographics that the guide could cater to. The lower the grade level, the larger the spectrum of demographics the guide could help [10].

Secondly, the similarity of the content was calculated using the QuillBot plagiarism tool (QuillBot, Chicago, IL), which is designed to detect potential plagiarism in the writing by evaluating the writing against a comprehensive database of online content, academic papers, and other sources [11].

Thirdly, the reliability of scientific text was evaluated using the modified DISCERN score, offering a reliable means to assess the quality of written information on treatment choices for health problems [12]. Each question was rated on a five-point scale ranging from no to yes.

Data and statistical analysis

Subsequently, the data was exported to a Microsoft Excel sheet (Microsoft Corp.) for further analysis. Statistical analysis was performed using R version 4.3.2 (The R Core Team, R Foundation for Statistical Computing, Vienna, Austria). The responses generated by ChatGPT and Google Gemini were compared using an unpaired t-test, and the correlation between the ease score and the reliability score was assessed using Pearson’s correlation coefficient (rs). A p-value < 0.05 was considered to be statistically significant.

## Results

Table 1 presents the average and standard deviation of key characteristics in responses generated by ChatGPT and Google Gemini. On average, ChatGPT produced responses with a higher word count than Google Gemini; however, this difference was not statistically significant (401 vs. 535.3 words, p = 0.2817). Google Gemini had a higher ease score on average (46.47 vs. 40.83), but this difference was also not statistically significant (p = 0.7244). Additionally, there were no significant differences in sentence count (p = 0.3241) or average words per sentence (p = 0.9917) between the two models.

**Table 1:** Characteristics of responses generated by ChatGPT and Google Gemini *Unpaired t- test; P-values <0.05 are considered statistically significant.

Variables	ChatGPT		Google Gemini		P-value*
	Mean	Standard deviation	Mean	Standard deviation	
Words	535.3333	72.67	401.0000	159.21	0.2817
Sentences	41.6667	9.61	30.6667	13.65	0.3241
Average words per sentence	13.5000	4.61	13.5333	2.12	0.9917
Average syllables per word	1.8000	0.10	1.7333	0.25	0.7024
Grade level	10.9000	1.91	10.1333	3.75	0.7732
Ease score	40.8333	8.72	46.4667	23.20	0.7244
Similarity %	17.7667	7.10	6.6667	4.99	0.0988
Reliability score	2.6667	0.5773	2.6667	0.5773	1.0000

**Figure 1:**
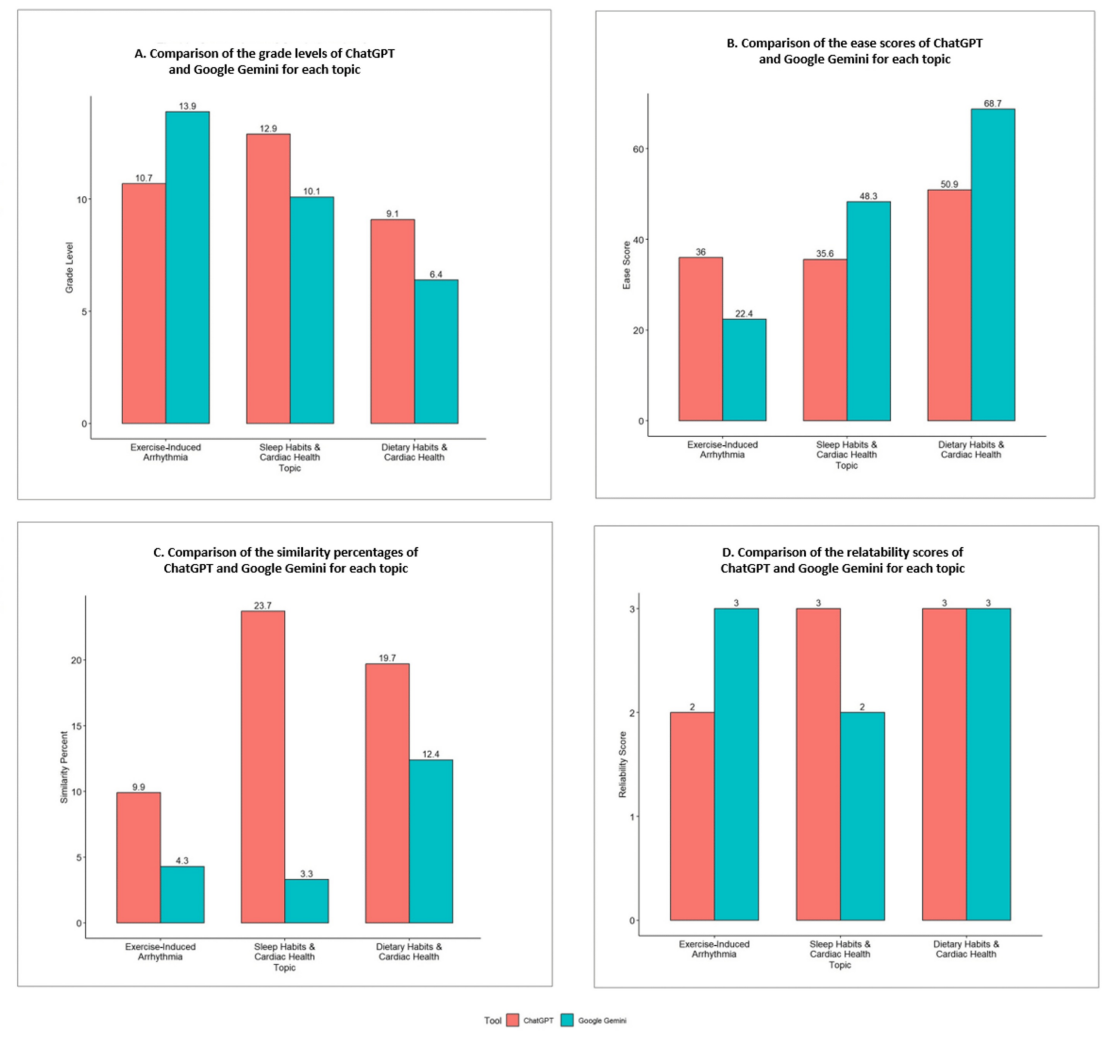
Graphical representation of the comparison between grade level (A), ease score (B), similarity percentage (C), and reliability score (D) for the patient education guide generated by ChatGPT and Google Gemini.

Figure 1 depicts the comparison between grade level, ease score, similarity percent, and reliability score for the patient education guides generated by ChatGPT and Google Gemini. According to the analysis of key characteristics for three topics related to cardiac health covered in Figure 1A, ChatGPT is inclined to generate content with a slightly higher complexity or grade level for dietary habits and cardiac health (12.9 vs. 10.1) and sleep habits and cardiac health (9.1 vs. 6.4). However, ChatGPT showed lower complexity for exercise-induced arrhythmia (10.7 vs. 13.9).

The ease score metric (Figure 1B), reflecting the readability or understanding difficulty, was higher for Google Gemini for the topics of sleep habits and cardiac health and dietary habits and cardiac health, with scores of 48.3 and 68.7, respectively, compared to ChatGPT's 35.6 and 50.9. Conversely, ChatGPT's content was rated easier to understand for the topic of exercise-induced arrhythmia, with a score of 36 compared to 22.4 for Google Gemini.

For similarity (Figure 1C), ChatGPT scored higher than Google Gemini for all three topics, i.e., for exercise-induced arrhythmia (9.9 vs. 4.3), sleep habits and cardiac health (23.7 vs. 3.3), and dietary habits and cardiac health (19.7 vs. 12.4).

Reliability scores (Figure 1D) were consistent for dietary habits and cardiac health, with each receiving a score of three, whereas, for the other two topics, the results show Gemini is more reliable for exercise-induced arrhythmia (three vs. two), and ChatGPT is more reliable for sleep habits and cardiac health topics (three vs. two).

## Discussion

The present study analyzed the patient education brochures about exercise-induced arrhythmia, sleep patterns and cardiac health, and dietary practices and cardiac health created by two artificial intelligence systems, ChatGPT and Google Gemini.

The comparison between responses generated by ChatGPT and Google Gemini for patient education guides on cardiac health topics provides valuable insights into the performance and suitability of these AI tools in providing health-related information. The present study observed that while ChatGPT produced responses with higher word counts compared to Google Gemini, indicating potentially more comprehensive content, this did not necessarily translate into better ease of understanding, as reflected by the ease score metric. Google Gemini consistently scored higher on ease of understanding across most topics, suggesting that its responses were more reader-friendly and accessible to a wider audience. However, it is important to note that ChatGPT demonstrated higher complexity in certain topics, which may be beneficial for addressing more specialized or in-depth aspects of cardiac health.

These disparities could be attributed to differences in the algorithms and training data of ChatGPT and Google Gemini. ChatGPT operates via extensive training on a wide range of texts and has been observed to generate detailed and comprehensive responses, leading to higher word counts [13, 14]. This could also be the explanation for the higher similarity percentage of ChatGPT compared to Google Gemini. On the other hand, Google Gemini has been observed to produce concise and easily understandable content [14, 15]. These differences can be attributed to the varying ease of understanding despite differences in word count.

Regarding reliability of content, the present study noted ChatGPT showing higher reliability in content generated for sleep habits and cardiac health, and Google Gemini exhibiting greater reliability for exercise-induced arrhythmia content. This could be attributed to the nature of the topics; Google Gemini, with access to real-time data and a vast array of online sources, may excel in providing information on relatively niche or uncommon topics, such as exercise-induced arrhythmia [15, 16]. In contrast, ChatGPT's responses may reflect the information it has been trained on, which could be broader but potentially less up-to-date or detailed on specific medical conditions [16].

El Sherbeni et al. found AI to have promising potential for applications in both primary and secondary cardiovascular disease (CVD) prevention, namely screening, detection, and tracking of risk variables [16]. Other studies have also emphasized the efficiency of AI in cardiovascular medicine due to its vast data vaults, increasing emphasis on precision care, and demand for operational efficiency. Ranka et al. have suggested future applications to include decision support for individual patient care of system-wide logistical activities as well as unique pathophysiologic findings [17]. However, the discrepancies in ease of understanding between ChatGPT and Google Gemini underscore the need for further refinement and optimization of AI algorithms to enhance readability and user comprehension [18]. Improving the readability of AI-generated content is crucial for ensuring that patients can easily understand and act upon the information provided, ultimately contributing to better health outcomes. Furthermore, future research should explore additional factors influencing the performance of AI tools in healthcare settings, such as the impact of different prompts or input formats on response quality and the potential role of human oversight in improving the accuracy and reliability of AI-generated content. Overall, while both ChatGPT and Google Gemini offer promising capabilities for generating patient education materials, careful consideration of their strengths and limitations is essential for maximizing their utility in healthcare communication and decision-making.

Limitations

Several limitations should be considered. Firstly, the comparison was limited to only two chatbots, ChatGPT 3.5 and Google Gemini, which may not represent the full spectrum of AI models available. Secondly, the focus on cardiovascular topics exclusively restricts the generalizability of the results to other medical domains. Thirdly, the utilization of ChatGPT 3.5, although convenient due to its accessibility, may introduce biases stemming from its reliance on pre-trained data, potentially leading to outdated or incomplete information. Additionally, Google Gemini’s reliance on real-time data access could introduce inconsistencies due to variations in online sources. The study also did not directly assess the accuracy of the chatbot-generated content, which would have provided valuable insights into their effectiveness in delivering medical information. Furthermore, readability and reliability were assessed separately, but the interplay between these factors was not fully explored.

Finally, the study did not account for potential differences in how these chatbots present information for different audiences; it can be hypothesized that Google Gemini’s responses could potentially be more accessible to patients with lower health literacy, while ChatGPT’s greater complexity could be more beneficial for clinicians or well-informed patients; future studies should expand the scope of chatbot models, analyze a broader range of medical topics, and assess both the accuracy and target suitability of AI-generated medical content.

## Conclusions

The comparison between responses generated by ChatGPT and Google Gemini revealed notable differences in word count and ease of understanding, with ChatGPT producing longer responses but Google Gemini scoring higher on ease of understanding. Our findings highlight that certain AI tools may be more suited for different applications: while tools like ChatGPT may be beneficial for detailed educational materials requiring depth, tools like Google Gemini could be more effective for layperson-friendly content due to their higher readability. A potential solution could involve integrating both models, leveraging ChatGPT for its depth of information and Gemini for its ease of understanding, providing a balanced approach to patient education.

Artificial intelligence’s ability to generate multilingual or culturally tailored patient education materials could significantly enhance its accessibility, addressing the diverse needs of global patient populations. It would also be valuable to investigate how different prompts affect AI output, as this could help optimize the models for specific educational goals. As AI continues to evolve, there is a need for consistent regulation and verification to ensure the accuracy and reliability of AI-generated content, and future research should focus on expanding the scope of AI tools, assessing their ability to generate high-quality patient education materials, and exploring their potential in various medical domains.
